# An Unusual Presentation of Tyrosinemia Type 1 in a Pediatric Patient: Case Report and Comprehensive Review

**DOI:** 10.1002/ccr3.70384

**Published:** 2025-04-01

**Authors:** Mahsa Rouhafshari, Mohammad Hadi Imanieh, Mahdi Khazaei, Zahra Radaei, Hamide Barzegar

**Affiliations:** ^1^ Department of Pediatric Gastroenterology Shiraz University of Medical Sciences Shiraz Iran; ^2^ Department of Pediatric Gastroenterology, Gastroenterohepatology Research Center of Nemazee Hospital Shiraz University of Medical Sciences Shiraz Iran; ^3^ Department of Internal Medicine Shiraz University of Medical Sciences Shiraz Iran; ^4^ Neonatal Research Center Shiraz University of Medical Sciences Shiraz Iran

**Keywords:** diaphragmatic paralysis, inborn errors, metabolism, tyrosinemia

## Abstract

Tyrosinemia type 1 often manifests with liver, renal, or peripheral neuropathy disorders. Before therapies like nitisinone, management was limited to dietary modifications and liver transplantation. We present a 19‐month‐old girl who developed respiratory distress requiring intubation, with abnormal laboratory findings, including liver function tests. Further work‐up, including succinylacetone testing, confirmed tyrosinemia. She responded remarkably to nitisinone treatment.

**Abbreviations**
5‐ALA: 5‐aminolevulinic acid
ALP: alkaline phosphatase
ALT: alanine aminotransferase
AST: aspartate aminotransferase
EEG: electroencephalogram
EF: ejection fraction
HT1: hereditary tyrosinemia type 1
LV: left ventricle
PICU: pediatric intensive care unit
PT: prothrombin time
PTT: partial thromboplastin time

**Summary**

- Tyrosinemia is an inherited metabolic disorder that can present with nonspecific features, including neurological crises such as respiratory distress, lethargy, or seizures, making early diagnosis challenging.
- However, it is rare for tyrosinemia to present with neurological symptoms as the first manifestation.
- Prompt recognition and treatment are essential to prevent severe complications or fatalities and improve patient outcomes.

## 1 Introduction

Tyrosinemia type 1 is a rare autosomal recessive inherited metabolic disorder caused by a deficiency in the enzyme fumarylacetoacetate hydrolase, leading to the accumulation of toxic metabolites such as fumarylacetoacetate and succinylacetone [1, 2]. Its clinical features primarily affect the liver, kidneys, and peripheral nervous system [3]. The most common presentations reported in the literature include failure to thrive, hepatosplenomegaly, bleeding, prolonged jaundice, family history screening, and abdominal distension [4, 5].

Since 1992, nitisinone has been introduced as an effective drug for reducing toxic metabolites by inhibiting 4‐hydroxyphenylpyruvate dioxygenase, making it a viable treatment option for tyrosinemia [6]. Before its availability, liver transplantation and dietary management were the only treatment options, and nitisinone marked a revolutionary advancement in therapy [7, 8].

Here, we present an infant with an unusual presentation who was diagnosed with tyrosinemia and responded very well to treatment.

## 2 Case History/Examination

A 19‐month‐old girl presented to the hospital with a 5‐day history of fever. She had no prior history of hospital admissions or medication use. Her parents were consanguineously married, and she had two siblings: a healthy 8‐year‐old and a 4‐month‐old sibling. There was no significant family history of illnesses.

On examination, the patient was lethargic. She weighed 10 kg (10th percentile), and her height was 70 cm (3rd percentile). Vital signs revealed a fever of 38.5°C, a respiratory rate of 55 breaths per minute, a heart rate of 130 beats per minute, blood pressure of 85/60 mmHg, and oxygen saturation of 91%–92%. She exhibited respiratory distress with retractions, tachypnea, and difficulty breathing. Abdominal examination revealed a firm hepatomegaly, with the liver palpable approximately 4 cm below the costal margin, and a slightly palpable spleen. Other findings were unremarkable.

## 3 Differential Diagnosis, Investigations, and Treatment

**FIGURE 1:**
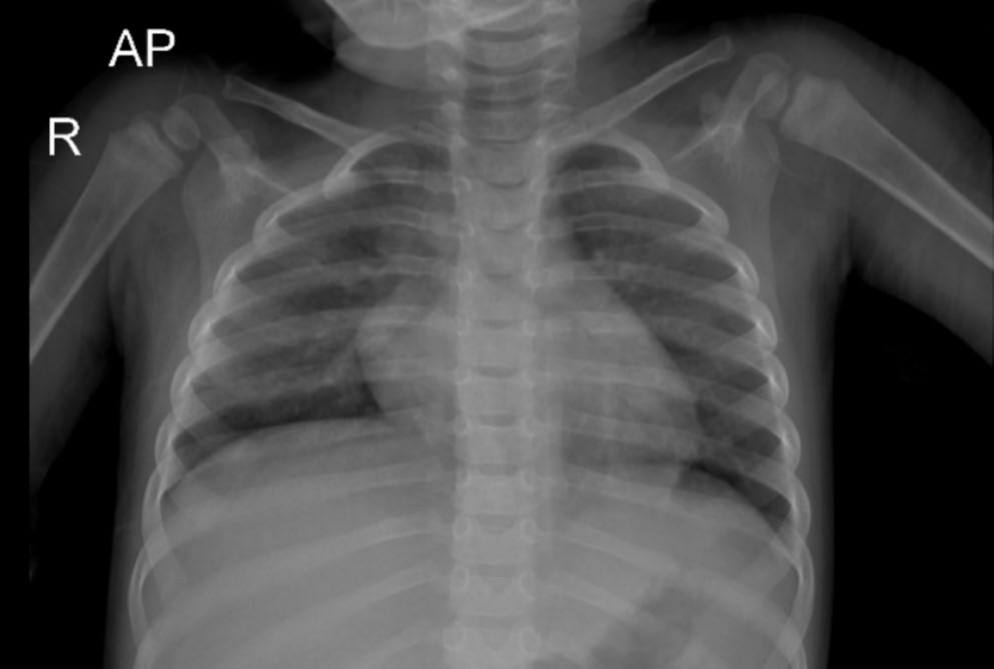
The anterior–posterior (AP) view shows clear lung fields without evidence of consolidation or infiltrates. The diaphragm appears slightly elevated, particularly on the right side, which may correlate with underlying hepatomegaly. The cardiac silhouette and rib alignment are unremarkable for this age.

The patient was admitted, and a series of diagnostic investigations were conducted to identify the cause of her symptoms. A chest X‐ray showed findings inconsistent with her severe respiratory distress (Figure 1). Echocardiography revealed mild left ventricular (LV) dilation with a preserved ejection fraction (EF) of 70%. Liver sonography demonstrated inhomogeneous parenchymal echogenicity with multiple high‐attenuation foci of varying sizes scattered across both liver lobes, with the largest measuring 4 × 3.5 cm. The spleen appeared inhomogeneous, and both kidneys exhibited increased parenchymal echogenicity. The brain imaging was normal, but the electroencephalography showed encephalopathy.

Laboratory data showed nothing in favor of sepsis but elevated liver enzymes and coagulation abnormalities. Initial findings included an AST of 230 U/L, ALT of 60 U/L, alkaline phosphatase (ALP) of 1570 U/L, prothrombin time (PT) of 43.5 s, partial thromboplastin time (PTT) of 86 s, and an INR of 3.4. Additional testing showed alpha‐fetoprotein levels over 2000 ng/mL, negative viral markers for hepatitis A, B, and C, ferritin of 171 ng/mL, ammonia of 167 μmol/L, and lactate of 19 mg/dL.

As her respiratory condition deteriorated, the patient was intubated and transferred to the Pediatric Intensive Care Unit (PICU). At this time, results from the urine succinylacetone test became available, revealing a significantly elevated level of 427.2 μmol/L, confirming a diagnosis of tyrosinemia. Nitisinone therapy was promptly initiated (1.5 mg/kg/day).

## 4 Conclusion and Results

She responded dramatically within 2 days, allowing for successful extubation, and all laboratory values normalized. She was subsequently discharged in stable condition on Nitisinone therapy (1 mg/kg/day).

Screening of her siblings revealed that her 4‐month‐old sibling also had tyrosinemia, though asymptomatic at the time.

This case highlights an atypical presentation of tyrosinemia in an infant with marked respiratory distress and hepatosplenomegaly. The patient showed a rapid and favorable response to Nitisinone, emphasizing the critical role of early diagnosis and intervention in managing this metabolic disorder.

## 5 Discussion

We present a case of an infant who exhibited neurological symptoms, including diaphragmatic paralysis leading to intubation, lethargy, and an abnormal EEG, ultimately diagnosed with tyrosinemia type I. Following diagnosis, the patient responded completely to treatment with nitisinone, underscoring the drug's effectiveness in resolving severe neurological manifestations associated with tyrosinemia.

In 1969, elevated levels of 5‐aminolevulinic acid (5‐ALA) were first documented in HT1 patients, attributed to the inhibition of 5‐ALA dehydratase by compounds such as succinylacetone. This disruption in the heme biosynthesis pathway leads to increased 5‐ALA levels, resulting in neurological crises similar to those observed in acute intermittent porphyria and lead toxicity [9, 10]. Management of neurologic crises primarily involves ensuring adequate caloric intake, providing respiratory support when necessary, and controlling pain [9].

HT1 generally manifests as liver dysfunction, kidney abnormalities, or other metabolic disorders. Neurologic crisis is rare as an initial presentation [11, 12]. Neurological symptoms, when present, are usually a consequence of acute metabolic crises or prolonged exposure to toxic metabolites. These episodes may lead to peripheral neuropathy, autonomic dysfunction, and, in severe cases, paralysis and respiratory failure. However, such symptoms are more commonly observed in older children or adolescents, often associated with inadequate treatment adherence or disease progression [2, 12, 13]. Neurologic crises are typically observed following the discontinuation of nitisinone [14]. There are reports of neurological crises in HT1 patients following the discontinuation of nitisinone. Mungan et al. documented a case of a 9‐month‐old girl with HT1 who experienced convulsions, diaphragmatic paralysis, respiratory distress, and self‐mutilation after 1 month without nitisinone [12]. Schlump et al. also reported an 8‐month‐old infant who presented with progressive ascending polyneuropathy, diaphragmatic paralysis, arterial hypertension, and respiratory distress after a 2‐month discontinuation of nitisinone. He was successfully treated with nitisinone [15]. In a 20‐year, single‐center study in Turkey, three patients experienced neurological crises due to medication disruption [16]. A rare presentation was reported in 2016 involving a 6‐year‐old girl diagnosed with tyrosinemia at age 2. She presented with acute, progressive abdominal pain, which was identified as pancreatitis. On the second day of admission, she developed a tonic–clonic seizure along with a slowing background on EEG. It was later discovered that her parents had discontinued nitisinone treatment for 6 months, leading to a neurological crisis [11]. As a result, it is essential to educate parents about the serious consequences of discontinuing nitisinone, emphasizing the risk of severe adverse effects, including neurologic crises.

Although most case reports support the hypothesis that neurologic crises typically occur following the discontinuation of nitisinone, our case presented a unique challenge, with neurologic symptoms appearing as the initial presentation. This unusual presentation underscores the importance of including tyrosinemia type 1 in the differential diagnosis to ensure timely intervention and prevent potentially fatal outcomes.

Screening for tyrosinemia type 1 can be achieved through tandem mass spectrometry, with succinylacetone—rather than tyrosine—serving as the pathognomonic biomarker for Tyrosinemia type 1 [17, 18]. Screening for tyrosinemia type 1 is available in many countries and can significantly reduce the risk of severe complications associated with atypical presentations, which may otherwise lead to life‐threatening outcomes before an accurate diagnosis is made.

In conclusion, this case highlights an atypical presentation of tyrosinemia type 1, where neurologic symptoms were prominent from the onset. Early recognition and diagnosis allowed for the timely administration of nitisinone, leading to a rapid clinical recovery. This underscores the importance of considering tyrosinemia type 1 in the differential diagnosis of unexplained neurologic crises in young children, especially those with abnormal liver function. Ensuring parental awareness about the potential consequences of discontinuing nitisinone is crucial for ongoing management and prevention of severe complications.

## Author Contributions

**Mahsa Rouhafshari:** conceptualization, project administration, writing – original draft, writing – review and editing. **Mohammad Hadi Imanieh:** conceptualization, project administration, writing – original draft, writing – review and editing. **Mahdi Khazaei:** data curation, writing – original draft, writing – review and editing. **Zahra Radaei:** data curation, writing – original draft, writing – review and editing. **Hamide Barzegar:** conceptualization, supervision, writing – original draft, writing – review and editing.

## Ethics Statement

The study protocol confirmed to the ethical guidelines of the 1975 Helsinki Declaration. The publication of this case was approved by the ethics committee of Shiraz University of Medical Sciences (IR.SUMS.MED.REC.1403.675). We have written informed consent obtained from the parents of the patient for the publication of this case report.

## Consent

We have written informed consent obtained from the parents of the patient for publication of this case report.

## Conflicts of Interest

The authors declare no conflicts of interest.

## Data Availability

All data generated or analyzed during this study are included in this published article.
